# Cross- and Within-Domain Associations of Early Reading and Mathematical Skills: Changes Across the Preschool Years

**DOI:** 10.3389/fpsyg.2021.710470

**Published:** 2021-10-12

**Authors:** Fu Yu Kwok, Rebecca Bull, David Muñez

**Affiliations:** ^1^Macquarie School of Education, Macquarie University, Sydney, NSW, Australia; ^2^Centre for Research in Child Development, National Institute of Education, Nanyang Technological University, Singapore, Singapore

**Keywords:** mathematics, reading, phonological awareness, preschool (kindergarten), longitudinal, number sets

## Abstract

Existing research has mainly examined the role of cognitive correlates of early reading and mathematics from a stationary perspective that does not consider how these skills unfold and interact over time. This approach constraints the interpretation of cross-domain associations and the *specificity* of domain-specific covariates. In this study, we disentangle the role of these predictors and investigate cross-domain associations between reading, math, and two related domain-specific predictors (phonological awareness and fluency with number sets) over the kindergarten years (*n*=512, Mage=54months, SDage=3.5, 52% females). Results reveal that the overlap between reading and math skills changes over development. Reciprocal associations between reading and math abilities are observed at earlier stages; then, reading abilities become the lead force. Findings also show that phonological awareness and fluency with number sets are domain-specific predictors that do not contribute to cross-domain gains in academic skills. Indeed, there is a trend for domain-specific skills to be more strongly related to achievement at the beginning of formal education than at the beginning of kindergarten, which suggests an increasing differentiation of domains over the kindergarten years. Such findings have implications for the timing and nature of interventions that aim to support children’s reading and mathematical development.

## Introduction

The acquisition of basic reading and numerical skills prior to school is argued to be the bedrock for continued learning as children enter formal schooling. In an influential study that investigated several datasets from different large-scale studies, Duncan et al. (2007) found that early math and reading skills have the greatest predictive power in school readiness and later achievement. Indeed, beyond allowing engagement in formal academic learning, such skills are predictive of longer-term life outcomes, including income, leadership in critical occupational roles, and even life expectancy (Ritchie and Bates, 2013; Lubinski et al., 2014). Decades of research on the acquisition and development of reading and math skills have shaped policy recommendations regarding instructional practices, materials, and assessment of reading and math skills at both national and international levels. Nonetheless, the bulk of evidence supporting such policy recommendations comes from domain-specific studies that have focused on either math or reading. This study aims to extend the existing knowledge base by exploring cross-domain associations between reading, math, and two related domain-specific predictors (phonological awareness and fluency in identifying and processing quantities represented by numerals and object sets) over the kindergarten years. Clarifying the possible existence of bi-directional associations on the underlying influences of reading and mathematics ability in early childhood may carry significant implications in the refinement of pedagogy and policy.

### Co-development of Reading and Mathematics

There is clear evidence that math and reading abilities are closely related (Davis et al., 2014). Findings from correlational studies suggest that about 40–50% of the variance in reading and math is shared. This association between reading and mathematics emerges during early childhood (McClelland et al., 2007) and persists into the elementary school years (Hecht et al., 2001). The comorbidity between difficulties in reading and mathematics also highlights the strong link between the development of reading and mathematical skills (Landerl and Moll, 2010; Mann Koepke and Miller, 2013; Moll et al., 2015, 2019). Although the origins and reasons for comorbidity are not clear yet, rates of comorbidity of reading and mathematics difficulties have been found to be more than 10 times larger than if they were unrelated conditions (Wilson et al., 2015). Consistent with this, a recent meta-analysis reported that children with a mathematical disability were approximately two times more likely to possess a reading disability compared to children without a mathematical disability (Joyner and Wagner, 2020 for a review).

This reciprocity has been investigated in non-experimental studies that have looked at how reading and math abilities jointly unfold and affect each other during childhood, through intervention studies, and through neuroimaging and behavioral studies examining the co-occurrence of math and reading difficulties. While the findings are arguably consistent in highlighting the cross-domain association between reading and mathematics, some differences exist in terms of methodological approaches and directionality of effects. For instance, an examination of six longitudinal data sets by Duncan et al. (2007), found early math skills to be a stronger predictor of later reading achievement, compared to early reading in its ability to predict math proficiency. More recent work by Bailey et al. (2020) draws further attention to the possible impact of the choice of analytical method on the cross-domain estimates obtained. In their study, the use of the traditional cross-lagged panel model yielded results similar to Duncan et al. (2007); in contrast, use of more recently developed variations of this model—which account for potentially confounding unmeasured individual and environmental factors—yielded attenuated estimates of the cross-domain association between reading and mathematics, showing evidence of low (but larger) paths from reading to later math (see also, Erbeli et al., 2020). Intervention studies also add to the evidence for reciprocal cross-domain associations. Improvements in mathematics ability have been observed following reading-oriented learning activities (Purpura et al., 2017). Similarly, engagement with structured learning activities designed to facilitate acquisition of mathematics skills has been found to predict stronger language ability in preschool (Sarama et al., 2012; Napoli and Purpura, 2018; but see, Fuchs et al. (2013), for differential findings).

Findings from neuroimaging studies also suggest a substantial overlap between reading and math. Activation of the left inferior frontal gyrus has been observed during the performance of both reading- and mathematics-related tasks (Andin et al., 2015). The phonological network, involved during the performance of reading-related tasks, has also been found to be activated during tasks requiring direct retrieval of mathematics-related facts or procedures (Li et al., 2019). Parts of the left temporo-parietal cortex—in particular, the left angular gyrus—have been linked with both reading (e.g., Pugh et al., 2001; Schlaggar and McCandliss, 2007) and math (e.g., Dehaene et al., 2003). Studies that have looked at neural activations in children with different patterns of difficulties in reading and math suggest that such comorbidity of reading and math deficits may be explained by neural underpinnings. Recently, Peters et al. (2018) explored the neural activation in four distinct groups of children (dyslexia, dyscalculia, comorbid dyslexia/dyscalculia, and typically developing) during reading and arithmetic tasks. They found that children with dyslexia, dyscalculia, and comorbid dyslexia/dyscalculia had similar neural activation patterns.

Two main hypotheses have been put forward regarding cross-domain associations between reading and math. From a functional perspective—or how abilities in one domain contribute to development in another domain—it has been suggested that this association can be attributed to the role of reading skills as the medium by which mathematics skills are acquired (Hecht et al., 2001; Jordan et al., 2003; Fuchs et al., 2005, 2006; Shin et al., 2013). In a similar vein, Cameron et al. (2019) posited that the co-development of reading and mathematics might be explained by cognitive processes that are involved in both abilities; they noted that symbol recognition is particularly important for performing reading and mathematics tasks at the preschool and kindergarten levels. Indeed, numeral literacy or learning to associate the well-known sounds of spoken language with symbol numbers—i.e., symbol-speech sound correspondence—does not involve different cognitive architectures for letter and number naming (Vander Stappen and Reybroeck, 2018). In other words, accessing the ordinal and cardinal meaning of number words/symbols is not required. For instance, neuroscience evidence shows that children aged 5years who can easily name letters and numbers, but who have not yet learned to read and operate with numbers at school, do not exhibit a neural dissociation between numbers and letters (Cantlon et al., 2011).

A complementary hypothesis is that co-development of reading and math abilities is explained by shared underlying factors (i.e., domain-general factors; Ferrer and McArdle, 2004; Purpura et al., 2019). This hypothesis does not preclude whether reading affects the development of math skills (or *vice versa*). Among the factors that may explain such co-development, there are several cognitive aspects (e.g., executive functions and intelligence). Cattell’s (1987) investment theory has also provided support to this hypothesis—a general cognitive factor would underlie the development of academic outcomes. For instance, in a recent meta-analysis of the relationship between academic achievement and broad abilities of the Cattell-Horn-Carroll theory, Zaboski II et al. (2018) found that the mean effect size of that general cognitive factor across all achievement domains and ages was r^2^=0.54. Other non-cognitive aspects, such as socioeconomic status, are known to affect both math and reading skills as well as the development of general cognitive abilities (e.g., Peng and Kievit, 2020).

Collectively, developmental, longitudinal, and neuroimaging studies provide distinct but converging evidence of the co-development of reading and mathematics before the formal schooling years. These findings underscore the importance of studying predictors of the development of both these abilities together rather than separately, to achieve a holistic understanding of how these skills mature during the early childhood years.

### Domain Specificity of Reading and Mathematics Predictors

Despite evidence of the co-development of reading and mathematics, research on early life predictors of these abilities has largely focused on examining them separately from each other. Notwithstanding, a handful of recent studies has examined the cross-domain overlaps between cognitive skills previously thought to be associated specifically with reading or mathematics ability at the preschool age. Some findings highlight the specificity of these within-domain associations. For example, Geary (2011) found that processing speed and the central executive component of working memory were predictors of both math and reading performance—domain-general predictors—with additional factors accounting for unique variance in each domain (i.e., phonological loop for reading, fluency in combining the cardinal value of collections of objects with the cardinal value of Arabic numerals for math)—domain-specific predictors. Similarly, Amland et al. (2021) and Fuhs et al. (2016) found that kindergartners and first graders’ phonological memory and general language competencies predicted later reading but not arithmetic achievement.

Other studies highlight evidence of cross-domain impact of these “domain-specific” skills. For instance, phonological awareness explains a substantial amount of the variance in math during the first years of formal education (e.g., Krajewski and Schneider, 2009; De Smedt et al., 2010; Zhang and Lin, 2018; Child et al., 2019; Vanbinst et al., 2020). Studies that have specifically modeled the shared variance between reading and math have reported similar cross-domain associations. These studies have distinguished a set of core predictors, such as non-verbal reasoning, working memory, and processing speed, as well as several cross-domain influences between domain-specific aspects that have been traditionally associated to either reading or math. For instance, it has been found that both counting skills and letter knowledge (which are broadly acknowledged as domain-specific predictors of math and reading, respectively) account for the shared variance in reading and arithmetic fluency (Korpipää et al., 2017; Koponen et al., 2018). In another study exploring domain-specific and domain-general predictors of reading and math, symbolic naming, phonological awareness, and rapid automatized naming were found to predict both reading and mathematics skills in kindergarteners (Cirino et al., 2018).

It has been postulated that conversion of numbers and operators into a verbal code is the first step in solving mathematics problems (Dehaene, 1992; Hecht et al., 2001). A child must first transform the numbers and operators in the problem into a speech-based code to solve both simple and complex mathematics problems (Dehaene, 1992; Hecht et al., 2001). Following this Arabic-to-verbal conversion, the child must then process the phonological information using a specific task-solving strategy by retrieving the answer directly from long-term memory; the ability to solve such a problem is dependent on the storage of phonological information (Amland et al., 2021). Finally, the phonological system may also be employed when the child uses the phonological codes for the number names in counting. In summary, there are several ways in which phonological processing (and phonological awareness, in particular) may yield a causal influence on math. Phonological processing is likely important for both decoding and arithmetic since both tasks depend on mental processes that use sound-based representations. Interestingly, children with better working memory—a cognitive skill that is thought to affect both reading and math—also show better phonological awareness (e.g., Alloway et al., 2005). In other words, as suggested by Chu et al. (2016), “*domain-general cognitive and learning systems will influence the acquisition of domain-specific knowledge and thus may be correlated with achievement in unrelated domains*” (p. 3).

While perhaps a less obvious directional association, a similar scenario can be found in relation to the association between precursors of math abilities and reading skills. For instance, Chu et al. (2016) found that kindergarteners’ sensitivity to the relative quantities of collections of objects and cardinal knowledge was predictors of reading skills. Counting skills are also among the precursors of math that usually correlate with reading skills (e.g., Koponen et al., 2013). Likewise, symbol recognition—a domain-specific predictor of mathematics ability—is significantly associated with reading development (Zemlock et al., 2018). From a functional perspective, it has been speculated that these skills contribute to strengthening visual-verbal associations in long-term memory, which are relevant for reading (Koponen et al., 2013). Nonetheless, as mentioned above, it is also possible that such association reflects the prior influence of domain-general systems and not the importance of content-specific knowledge *per se—*i.e., domain-specific skills as a proxy for individual differences in domain-general abilities that predict achievement across academic domains (Chu et al., 2016).

Taken together, these findings suggest that domain-specific predictors are not so specific in the sense that they are not uniquely associated with reading (or math) and that similar cross-domain associations (to those observed between math and reading) may emerge at the level of domain-specific predictors. Given the degree of association between same-domain variables (e.g., phonological awareness and reading) and that of reading and math skills, it is not surprising that cross-domain associations between domain-specific predictors and reading (and math) have been observed. Arguably, if an underlying factor explains variability in math and reading, then, the same factor should explain the development of other math- and reading-related aspects. Furthermore, although the magnitude of cross-domain associations between domain-specific skills and math and reading has been used to differentiate between prior influences of domain-general aspects and the influence of content-specific knowledge *per se*, it is not clear whether those cross-domain associations simply reflect differences in academic domains—reading and math (e.g., phonological awareness as a proxy for reading skills and counting skills as a proxy for math abilities).

Extant findings are inconclusive and there are multiple factors that may alter the *specificity and reciprocity* of domain-specific variables (e.g., adequacy of measures and control of confounding variables, research design, developmental stage, and methodological approach). For instance, the literature suggests that cross-domain associations of domain-specific skills and reading and math are more likely in early childhood. This is because domain-general aspects (i.e., a common underlying factor that also contributes to development in domain-specific predictors of reading and math abilities) are more relevant in younger children’s reading and math. For instance, in a longitudinal study with children from first to eighth grade, Geary et al. (2017) found that the role of domain-specific knowledge on math increased over development and that the contribution of domain-general aspects was stronger for younger children’s mathematics. This does not mean that within-domain associations are weak at earlier stages in development but that domain-specific aspects are not yet differentiated. For instance, correlations between different early numeracy skills in children (e.g., verbal counting, numeral identification, subitizing, number comparison, and number order) are usually moderate to high. Indeed, studies that have specifically investigated the factor structure of measures of early numeracy skills either have failed to identify more than one factor or have reported factor structures that are controversial due to high correlations between factors (e.g., Braeuning et al., 2020; Purpura and Lonigan, 2013). Similarly, the strength of the associations between skills related with early reading ability also suggests a higher degree of overlap in younger children (Schatschneider et al., 2002; Vukovic and Siegel, 2006). For instance, Poulsen et al. (2015) found that phonological awareness explained a significant proportion of the variance of the association between rapid automatized naming and reading in a large sample of children followed-up from kindergarten to Grade 1. As such, the extent to which phonological awareness and rapid automatized naming can be differentiated from each other, and the exact contributions of each skill on reading development are inconclusive at best (Van der Ven et al., 2012). Collectively, these findings underscore the complexity of unraveling the influence of various cognitive processes involved in early development of reading and mathematics.

Furthermore, it is not surprising that reading skills contribute substantially to variance in math achievement (and *vice versa*) at earlier stages in development given the role of numeral literacy in the set of math skills that young children are expected to master. For instance, Braeuning et al. (2020) investigated the multifactorial structure of the ECLS-K math assessment with a large sample of preschoolers and found that the largest factor loadings of indicators representing number sense (one of four factors that was identified) corresponded to number knowledge items—e.g., identifying a written Arabic number. Indeed, this raises additional questions regarding the adequacy or specificity of math and reading measures for younger children; is number naming different from letter naming in children who have not yet learned to read and operate with numbers at school?

### Present Study

The purpose of the present study is to further elucidate the role of two domain-specific predictors that have been broadly investigated in the literature—phonological awareness, one of the strongest cognitive correlates of learning to read (Melby-Lervåg et al., 2012) and of disabilities in reading (Snowling, 2001), and children’s fluency in identifying and processing quantities represented by numerals and object sets, which is associated with math achievement (e.g., (Geary et al., 2007, 2009; Fuchs et al., 2010a,b; Geary, 2011), and may serve to identify children with mathematical learning difficulties (Geary et al., 2009). Specifically, in a path model, we examine whether these are indeed domain-specific predictors, or whether they show cross-domain associations (i.e., they represent shared cognitive correlates of reading and math development). Secondly, the study also examined the cross-domain association between early reading and mathematics. Lastly, the bi-direction longitudinal associations between reading and mathematics from preschool (age 5) to the first year of formal education (age 7) were examined.

## Materials and Methods

### Participants

Data from the current study were drawn from a large-scale longitudinal study examining the impact of preschool education on children’s development in Singapore (Singapore Kindergarten Impact Project; Ng and O’Brien, 2020). Recruitment for the main study followed a stratified sampling strategy to target mainstream preschool centers from a range of social strata. The sample for the current study was selected based on testing window (February to April of K1) and testing interval (12months between each data collection point), resulting in a final sample of 512 children (M_age at K1entry_=54months, SD=3.5; 52% females). In terms of ethnicity, 324 children identified as Chinese, 59 as Malay, 94 as Indian, and 18 as others (17 children did not have ethnicity information). All children were attending kindergarten which provides half-day (2.5–4h) care and education in the 2years prior to formal schooling. Approximately 97% of children attend at least 1year of preschool (kindergarten or full-day childcare) education (Bull and Bautista, 2018). The “Nurturing Early Learners” (NEL) Kindergarten Curriculum Framework sets out key knowledge, skills, and dispositions that children are expected to demonstrate by the end of kindergarten. For literacy, this includes demonstrating print awareness, alphabet knowledge, phonological awareness, and recognizing familiar and high frequency words (Ministry of Education, 2013). For early numeracy, skills include recognizing and using simple relationships and patterns, number recognition, counting to 10, understanding of counting principles, comparing quantities, representing quantity in different format and transcoding between them, part-whole relationships, shape recognition and manipulation, and use of position, direction, and distance referents (Ministry of Education, 2013). Literacy and numeracy are just two of six curriculum areas, and there is no specified amount of curriculum time that educators are expected to dedicate to literacy and numeracy activities.

### Procedure

Data collection was done as part of a larger study, which included other measures apart from those utilized in the present study. Each task was administered individually to the child. Total administration time per child for the larger study ranged from 4 to 5days. In the current study, time-invariant measures (SES, non-verbal intelligence) were collected at entry to K1 and included as covariates in the analyses. We also controlled for age differences in K1 measures. For the remaining measures, children were tested at entry to K1 (the year children turn 5), K2 (the year children turn 6), and P1 (the year children turn 7).

### Materials

#### Reading Skills: Wide Range Achievement Test—4th Edition

The *Word Reading* subtest was used to measure children’s early literacy skills (Wilkinson and Robertson, 2006). It consisted of Letter Reading (15 items) and Word Reading (55 items). Only the Green form was administered for letter reading, whereas both the Green and Blue forms (parallel versions that can be used interchangeably with comparable results) were administered for word reading; the Green form was always administered first. Test items were scored as “1” if children read the letter/word correctly. Only the word reading task had a discontinue rule, whereby test administration was terminated after 10 consecutive incorrect responses. A reading score was derived by summing the average of the child’s word reading score on the Green and Blue forms with the letter reading score (i.e., mean [Green and Blue word reading]+letter reading). A high score indicates better reading skills. Test-retest reliability values using K1 and K2 data were good (ICC and 95% CI)^1^ = 0.91 (0.90, 0.93).

#### Math Skills: Test of Early Mathematics Ability—3rd Edition

This task measures children’s informal and formal mathematics knowledge (Ginsburg and Baroody, 2003). Informal knowledge (acquired outside the context of schooling) is measured through four categories of items: numbering (e.g., verbal counting by ones), number comparisons (e.g., choosing the larger number), calculation (e.g., addition of concrete objects), and concepts (e.g., number constancy). Formal knowledge (skills and concepts learned in school) is also assessed *via* four categories: numeral literacy (e.g., reading or writing numerals), number facts (e.g., subtraction facts), calculation (e.g., written addition accuracy), and concepts (e.g., written representation of sets). The dependent measure was the number of items answered correctly. Items in each of the categories increased in difficulty level as children progress further in the task. Following the TEMA-3 manual, test administration began with an entry point suitable for the children’s age and was terminated when ceiling (five items incorrect in a row) and basal (five items correct in a row) were established. Then, we scored all items below the basal correct and all items above the ceiling incorrect. A high score reflects better math skills. Test-retest reliability values using K1 and K2 data were good (ICC and 95% CI)^1^=0.92 (0.91, 0.93).

#### Phonological Awareness: Comprehensive Test of Phonological Processing—2nd Edition

Two subtests from the Comprehensive Test of Phonological Processing—2nd Edition were used to measure children’s phonological awareness (Wagner et al., 2012). In the *Elision* subtest (34 items), children were required to listen to a word (e.g., *cup*), repeat it, and then say what is left of that word after dropping designated sound segments (e.g., /k/; *up*). Corrective feedback was given on the first 14 items. The *Blending Words* subtest (33 items) required children to listen to a series of audio-recorded words spoken in segments (e.g., /t/ and /oi/) and to reproduce the whole word (e.g., *toy*) by blending the sound segments. Corrective feedback was given on the first 12 items. A phonological awareness score was derived by summing the total scores from both subtests (i.e., Elision + Blending). A low score indicates low phonological awareness. Test-retest reliability values using K1 and K2 data were good (ICC and 95% CI)^1^=0.88 (0.87, 0.90).

#### Fluency in Identifying and Processing Quantities Represented by Numerals and Object Sets: Number Sets Test

This task assessed the speed and accuracy with which children can identify and process quantities represented by Arabic numerals and/or object sets in a paper-and-pencil format (Geary et al., 2007). Children were presented with pairs or trios of objects (e.g., ▲▲▲|▲▲), Arabic numerals (e.g., 2|3), or both (e.g., 4|▲, ●●|2|▲). Each combination pair or trio is considered an item. Children were required to circle items that matched a target number (five or nine) quickly and accurately within a given time limit (60s for target number “five”; 90s for target number “nine”). Performance on this task depends on children’s ability to subitize and map Arabic numerals into representations of small quantities and to perform simple addition with small sets and Arabic numerals (Rousselle and Noël, 2007). The following information was collected from children’s responses: the number of items correctly identified as matching the target number (hits), the number of correct matches that were not identified (misses), the number of incorrect items that were identified as matching the target (false alarms), and the number of incorrect items that were not identified (correct rejections). We used a sensitivity measure, d-prime (z scores for hits – z scores for false alarms; MacMillan, 2002), as the performance measure. Test-retest reliability values using K1 and K2 data were good (ICC and 95% CI)^1^=0.84 (0.82, 0.87).

#### Non-verbal Intelligence

The Ravens Colored Progressive Matrices were used as a measure of children’s non-verbal reasoning ability (Raven, 1947). The dependent measure was the total number of correct responses across all three sets. Higher scores reflect better non-verbal reasoning ability. Internal consistency in the whole sample was good (*α*=0.89).

#### Socioeconomic Status

A composite SES score was derived from a principal component analysis of four variables: mother’s education, father’s education, family income, and housing type. Housing type is a common indicator of SES in the Singapore context (e.g., Sabanayagam et al., 2007).

## Analyses

To investigate the substantive questions in the current study, we formulated a path model with four different longitudinal chains corresponding to math, reading, phonological awareness, and fluency with number sets. In this model, autoregressive paths (i.e., the extent to which scores at time t for a variable X affect scores at time t+1 for the same variable) were constrained to equality to reflect similar associations between each pair of adjacent measurements because the intervals between time points were similar (about 12months). Cross-lagged relations between different variables (i.e., the extent to which scores at time t for a variable X affect scores at time t+1 for a different variable) were freely estimated to reflect different cross-domain associations over time. Residual variances at each time point were correlated. In this model, we also included time-invariant covariates (SES and non-verbal intelligence) that are known to affect both reading and math skills as well as related domain-specific predictors. These covariates (domain-general predictors) were linked to all variables of interest (reading, math, and related domain-specific predictors) and time points in the path model. We also controlled for age differences at entry to kindergarten since the development of the skills that were measured likely starts before the onset of preschool education. For instance, the odds that older children have better language and numerical skills than younger children are higher.

Parameters were estimated with full information maximum likelihood. Model fit was assessed by inspecting the *χ^2^* test, as well as the Comparative Fit Index (CFI; values above 0.95 indicate adequate fit, Hu and Bentler, 1999), Root Mean Square Error of Approximation (RMSEA; values below 0.06 indicate good model fit, Chen et al., 2008), and SRMR (values <0.08 indicate good fit, Hu and Bentler, 1999). We used a robust maximum likelihood estimator (MLR), with standard errors that are robust in relation to non-normality and non-independence of observations. All analyses were conducted using MPlus version 8.6 (Muthén and Muthén, 2017). Although some variance lies at the classroom level, our model did not take into account classroom level clustering because of student mobility across the grades. Furthermore, at Kindergarten 2, there were a large number of clusters (87) with a small average cluster size (5; range 1–15).

## Results

Descriptive statistics and age-adjusted bivariate correlations are shown in Table 1 (top and bottom panel, respectively).

**Table 1:** Descriptive statistics (top) and age-adjusted bivariate correlation table (bottom).

	Math1	Math2	Math3	Read1	Read2	Read3	Nset1	Nset2	Nset3	Phaw1	Phaw2	Phaw3	SES	NvIn
n	498	508	507	505	509	508	510	511	507	503	508	506	467	487
M	23.31	34.52	44.69	14.77	21.55	29.64	0.45	1.4	2.12	13.48	22.33	30.08	−0.08	14.59
SD	8.96	9.24	10.12	5.29	7.24	8.02	0.56	0.72	0.68	7.66	9.52	8.99	0.99	4.66
Skewness	0.14	0.19	0.57	0.00	0.79	0.38	0.9	−0.34	−0.81	0.66	−0.04	−0.23	−0.44	0.2
Min	0	8	16	0	1	15	−0.72	−0.39	−0.15	0	0	0	−2.81	2
Max	49	70	72	35.5	48	55	2.67	3.29	3.68	41	53	56	1.75	30
Math2	0.78	–												
Math3	0.65	0.77	–											
Read1	0.61	0.54	0.48	–										
Read2	0.60	0.59	0.53	0.75	–									
Read3	0.55	0.57	0.54	0.60	0.79	–								
Nset1	0.52	0.46	0.45	0.32	0.30	0.24	–							
Nset2	0.66	0.74	0.69	0.41	0.44	0.43	0.50	–						
Nset3	0.57	0.64	0.69	0.36	0.38	0.37	0.37	0.69	–					
Phaw1	0.57	0.45	0.43	0.43	0.49	0.48	0.36	0.40	0.34	–				
Phaw2	0.63	0.58	0.49	0.53	0.65	0.67	0.31	0.48	0.42	0.63	–			
Phaw3	0.50	0.48	0.48	0.42	0.53	0.63	0.21	0.41	0.39	0.52	0.69	–		
SES	0.33	0.36	0.34	0.29	0.35	0.32	0.16	0.32	0.24	0.35	0.43	0.32	–	
NvIn	0.42	0.39	0.35	0.29	0.28	0.30	0.33	0.43	0.37	0.33	0.33	0.28	0.24	–

*All correlations are significant at the level p<0.001. Math=TEMA raw score; Read=Letter and Word Reading raw score; NSet=Number Sets d-prime; SES=socioeconomic status standardized score; NvIn=Ravens Non-verbal Intelligence raw score; 1=K1 assessment; 2=K2 assessment; and 3=Grade 1 assessment*.

The within-variable correlations over time showed a typical autoregressive pattern (i.e., stronger correlations among observations taken in adjacent waves). This pattern was more evident for reading and math than for phonological awareness and number sets. Indeed, reading and math measures were quite stable over time and showed good reliability (above 0.75 for a one-year gap). Overall, cross-domain associations were large according to Cohen’s (1988) standards. Associations of domain-general variables with verbal and numerical variables were moderate and did not change substantially across time points.

### Path Model

**Figure 1:**
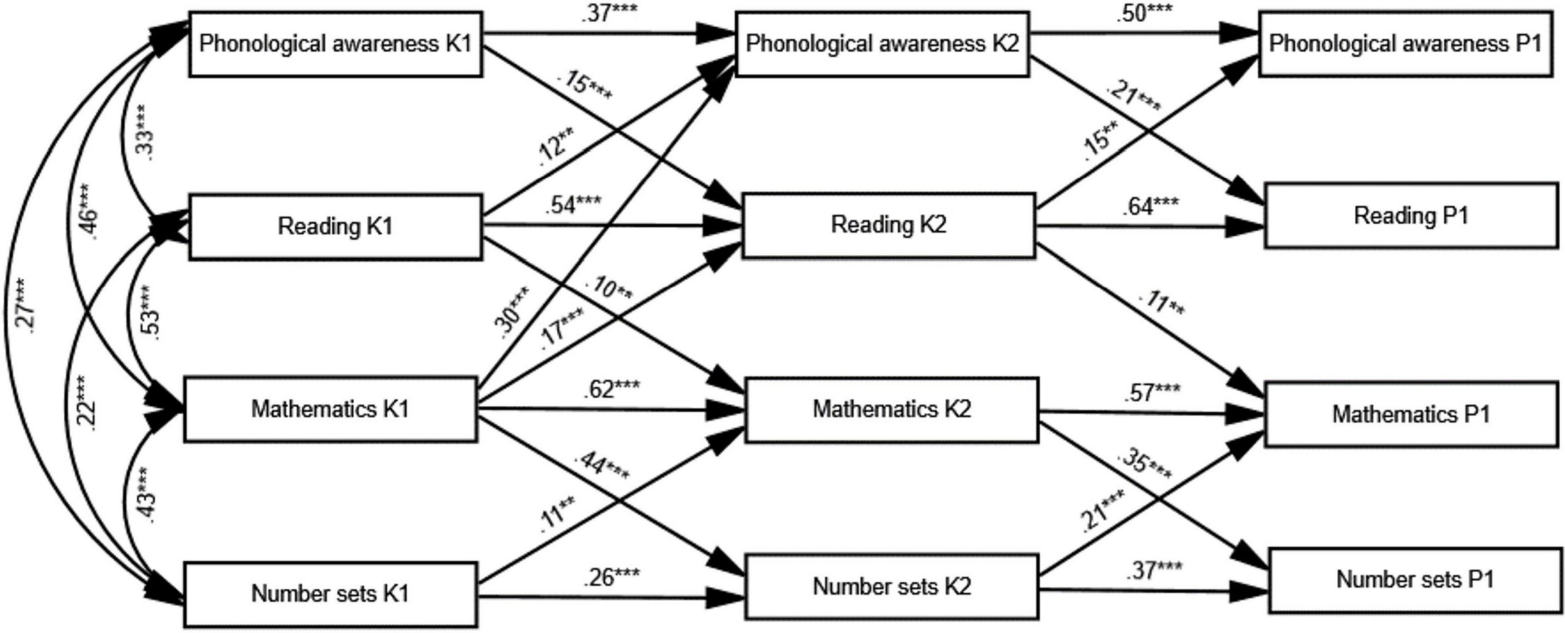
Path model with parameter estimates that only significant path is shown in the diagram. For clarity, paths from covariates (SES, age, and non-verbal ability) and residual co-variances are not shown. ^*^p<0.01 and ^*^^*^^*^p<0.001.

The model that was specified fitted the data well (*χ^2^* (28)=53.53, CFI=0.994, TLI=0.979, RMSEA=0.042 [0.025, 0.059]). Parameter estimates are shown in Figure 1. At entry to K1, children from higher SES backgrounds, those who were older, and those with better non-verbal reasoning skills also had better reading and math skills as well as better scores on number sets and phonological awareness (see parameter estimates in Supplementary Material; Supplementary Table S1). The proportion of explained variance differed across DVs—ranging from 17 to 30%.

At entry to K2, the analysis revealed SES disparities across the four variables, which indicates that the SES gap in cognitive development increased during the first year in kindergarten. Non-verbal intelligence also predicted scores on number sets. Note that the null association between non-verbal reasoning and the remaining K2 variables means that the magnitude of the disparities found at entry to kindergarten persisted in K2. The analysis also revealed cross-domain associations (or reciprocal influence) between reading and math after accounting for previous math and reading skills and the effect of domain-specific predictors. The standardized coefficients of the cross-lagged associations between reading and math suggest that math ability at entry to kindergarten may have a stronger role in the development of reading skills than that of reading skills in the development of math skills during the first year in kindergarten.

Each domain-specific predictor was associated uniquely with its corresponding same-domain variable. The magnitude of these domain-specific associations was similar to that of the cross-domain associations; math and phonological awareness had similar effects on reading skills at the beginning of K2 (0.167 and 149, respectively) and reading and number sets had similar effects on math skills at the beginning of K2 (0.101 and.108, respectively). The magnitude of those associations was small in terms of Cohen’s (1988) standards (equivalent to.10–0.17 SD). Note that these associations were statistically significant after accounting for individual differences in the same variable at an earlier time point, so they reflected associations with reading and math growth over the first year in kindergarten. It is worth mentioning that whereas fluency with number sets in K2 was related to math skills in K1 but not reading skills in K1, variability in phonological awareness in K2 was partly explained by math skills in K1. Indeed, this association was stronger than that with reading skills in K1; a 1 SD increase in math at K1 was associated with a 0.30 SD increase in phonological awareness at K2. In comparison, a 1 SD increase in reading at K1 was associated with just a.12 SD increase in phonological awareness of K2.

At entry to P1, only non-verbal reasoning remained associated with scores on the number sets task. This long-term association is noteworthy even if the magnitude of the effect decreases over time; children with better non-verbal reasoning skills at entry K1 showed more sustained gains over the 2years in kindergarten. It also underscores the non-verbal component of the skills required to solve the number sets task (see “Discussion”). As mentioned above, failing to observe an association with the remaining P1 variables (and the null association between SES and P1 variables) indicates that the magnitude of disparities related to domain-general aspects in K2 persisted when children entered formal education. The analysis also revealed a higher degree of disambiguation between reading and math skills. There were no reciprocal associations between reading and math; only reading skills at K2 were associated with math gains at entry to P1 (math at K2 was not associated with reading gains at entry to P1) and each domain-specific variable was related to its corresponding same-domain variable. The proportion of explained variance was similar across DVs at entry to K2 and P1 (about 50–55% for phonological awareness and fluency with number sets and slightly higher—60–65%—for reading and math skills).

It is worth mentioning that, although the role of the domain-specific predictors increased over time, the dynamics and reciprocal associations between domain-specific predictors and the corresponding academic outcomes (math or reading) were different across domains. Phonological awareness was the leading force in the development of reading skills (this was more evident during the last year in kindergarten). In contrast, the association between math abilities and children’s fluency with number sets was one in which math was the leading force over the kindergarten years. Furthermore, although the analysis revealed some cross-domain associations between reading and math over the kindergarten years, we did not observe any reciprocal association between domain-specific predictors.

## Discussion

This study examined the reciprocal associations between early reading and mathematics, and two domain-specific predictors—phonological awareness and fluency with number sets (the skill of operating with different numerical representations). We focused on elucidating the role of these domain-specific skills as well as the within- and cross-domain associations that emerge over the kindergarten years. Results from the study revealed three key findings. First, phonological awareness and number sets are domain-specific predictors of reading and mathematics, respectively (and do not contribute to cross-domain gains). Second, while there are cross-domain associations between reading and math (and between math and phonological awareness), these associations are not stable over time. Third, there are within-domain bi-directional associations between the more general skill (reading or math) and their respective domain-specific skills (phonological awareness and number sets). The pattern of findings suggests that cross-domain associations are more evident when it comes to reading and general math abilities and that the strength of within-domain associations increases over time (as the level of cross-domain associations decreases). We discuss each of these key findings in more detail below.

### Domain-Specific Prediction of Reading and Mathematics

Fluency with number sets and phonological awareness were associated with their respective domains (math and reading, respectively), uniquely contributing to growth in each domain. Although the effect size of those associations was small, it was fairly consistent across domains and increased over time. At entry to K2, the effect size was equivalent to about 0.13 SDs (in reading and math), whereas at entry to P1, that effect size increased to about 0.20 SDs. Note that those effects are calculated after accounting for reading and math disparities at previous stages, which underscores the relevance of phonological awareness and fluency with number sets on reading and math improvements, respectively. In contrast to some previous studies, we did not find that phonological awareness predicted later math achievement (Hecht et al., 2001; Fuchs et al., 2005; De Smedt et al., 2010; Child et al., 2019; Vanbinst et al., 2020). However, our results do align with findings from a recent meta-analysis (Peng et al., 2020) showing that weak relationships of phonological awareness to general math, numerical knowledge, calculations, and word problems were rendered non-significant after controlling for other domains general skills, in this case, working memory and intelligence (see also Amland et al., 2021; Bernabini et al., 2021; Pinto et al., 2016). It is noteworthy that in our model, the role of phonological awareness on later math achievement refers to the contribution to gains in math after accounting for differences in reading skills. Thus, it is feasible that such association reported in other studies simply reflects the role of phonological awareness as a proxy for reading skills or other aspects of phonological processing that may be involved in recognizing symbol numbers (e.g., RAN) or maintaining several chunks of information in memory during multi-step problem solving (short-term memory). These three aspects of phonological processing are highly correlated, and a single phonological processing dimension has been frequently posited (Wagner et al., 1987, 1993; Wagner and Torgesen, 1987).

Far fewer studies have considered whether early developing numerical skills, like those measured by the number sets task, predict reading as well as math. Vanbinst et al. (2020) found that only certain numerical skills (numeral recognition) predicted concurrent reading in 5-year olds (indexed by a letter knowledge task), while numerical magnitude skills (non-symbolic and symbolic comparison) showed no significant cross-domain association (see Child et al., 2019 for similar non-significant prediction of non-symbolic discrimination to reading skills in Grade 2 students). Cirino et al. (2018) in a longitudinal study of children from kindergarten to Grade 1 found that symbolic labeling (ability to name single, two, and three-digit numbers) predicted literacy outcomes (decoding, reading fluency, and reading comprehension). Other numerical measures (rote counting, counting knowledge, and symbolic comparison) did not predict literacy outcomes. In contrast, studies of slightly younger children (3–4years of age) show that both sensitivity to relative quantities and cardinal knowledge are associated with later reading skills (Chu et al., 2016). Such findings suggest that the prediction of emerging numeracy skills to later reading could be associated with the requirement to recognize abstract symbols, the ability to retrieve associations between visual symbolic and phonological forms, or general processing skills, such as visual attention, that may be common to letter learning and non-symbolic quantity discrimination skills (Anobile et al., 2013). As mentioned above, the same mechanisms that contribute to strengthening associations between sounds of spoken language and symbol numbers may contribute to letter-speech sound correspondence. Further research is needed to confirm the mechanism of this relation between early numeracy skills and later reading skills and the changing nature of this association with age and experience.

### Cross-Domain Associations Between Reading and Mathematics

The second main finding is generally consistent with existing literature supporting a cross-domain association between reading and mathematics (e.g., Duncan et al., 2007; Bailey et al., 2020). Nonetheless, cross-domain associations changed over development. Specifically, mathematics was found to predict growth in reading from wave 1 (Kindergarten 1) to wave 2 (Kindergarten 2) but not from wave 2 to wave 3 (Primary 1). In contrast, reading was a consistent predictor of growth in mathematics ability over the kindergarten years. Although the magnitude of this association was small, it was consistent over development. Erbeli et al. (2020) reported similar findings in elementary children from grades 1 to 4, where annual change in math growth was (partially) accounted for by reading achievement. The reverse coupling, annual change in reading growth predicted by math achievement, was not found. Our findings extend this down to children in the year prior to formal schooling. However, prior to this age, it appears that reciprocal coupling better depicts the development of reading and math skills. Our results also align with findings from Jordan et al. (2003) which indicated that reading performance influenced growth in math, but the reverse direction of influence was not evident. Specifically, elementary school children with math difficulties who were good readers showed greater growth in math compared to children who have both reading and math difficulties. No such advantage was seen for children with reading difficulties who had good math skills—they showed comparable growth in reading as those with comorbid difficulties.

Another observed cross-domain association was that mathematics at K1 predicted change in phonological awareness from K1 to K2. Indeed, the magnitude of this association was similar to that of phonological awareness at an earlier time point (equivalent to 0.30 SD in phonological awareness at entry to K2). A similar cross-domain association was not found for reading at K1 and number sets at K2. A possible explanation for this finding might lie in symbol recognition abilities. Letters of the alphabet and numbers have abstract representations in the form of sounds and quantitative values, respectively. Understanding a system of quantity-related symbols (numbers) may therefore help to facilitate learning a system of sound-related symbols (letters). In this way, symbol recognition may explain how math abilities can contribute to the subsequent development of skills related to phonological awareness. Purpura et al. (2017) also found that earlier math ability predicted growth in phonological awareness. However, this direct relationship was mediated by early math language skills. Others have argued that some math assessments involve both language and code-based skills, and hence may be a proxy for early language abilities, accounting for the prediction of concurrent or later reading abilities (Purpura and Napoli, 2015). In the current study, this may apply to the TEMA which includes skills, such as numeral literacy and counting fluency. In contrast, the number sets task has very little reliance on such language and code-based skills and was not found to predict later reading skills.

Although different hypotheses have been formulated to explain cross-domain associations between reading and math abilities, these hypotheses are complimentary and probably reflect different developmental stages. For instance, it is feasible that underlying shared factors contribute to a larger extent to reading and math at earlier stages. This would explain the association of math with later reading and verbal abilities (as well as that of reading with later math skills) during the first year in kindergarten. Then, content-specific influences may shape to a larger extent the cross-domain associations (for a similar explanation see De Smedt et al., 2010). Exposure to a more diverse set of math abilities during the last year in kindergarten or at the beginning of formal education probably increases the specificity of the mathematical and numerical domain. This aligns with findings from correlational studies that have looked at the role of domain-general aspects. It is thought that domain-general competencies become less relevant as children gain domain-specific expertise (Geary, 2005; Sweller, 2015). In other words, if cross-domain associations between reading and math rely on (domain-general) underlying factors; then, such associations are likely to vanish as the role of domain-general factors decreases.

### Within-Domain Bi-directional Associations

Phonological awareness and number sets do not appear to represent developing precursors to reading and math, respectively. Instead, we see evidence of within-domain bi-directional longitudinal prediction. For example, K1 number sets predict K2 math, but the reciprocal prediction from K1 math to K2 number sets is considerably larger (equivalent to about 0.40 SD in fluency with number sets, which is a moderate effect size in terms of Cohen’ standards; Cohen, 1988); a similar pattern of bi-directional associations is also seen from K2 to P1. We see a similar reciprocal relationship between phonological awareness and reading, although earlier phonological awareness to later reading is slightly stronger than from earlier reading to later phonological awareness. Within the mathematical cognition literature, there is ongoing debate regarding the directionality of association between basic number sense skills and formal mathematical ability. Our findings support the idea of a bi-directional relationship (see also LeFevre et al., 2013; Friso-van den Bos et al., 2015; Elliot et al., 2019), whereby basic skills, such as understanding quantity from symbolic and non-symbolic representations, and transcoding between and combining those representations predict more general math achievement. However, they are not precursors to math achievement, because at the same time, we find that general math achievement predicts growth (in this case accuracy and fluency) in using those basic skills. While a number of studies have found performance on the number sets task to predict growth in math achievement (e.g., Fuhs et al., 2016; Geary et al., 2017), these studies did not consider the possibility of a bi-directional relationship. In the field of reading, notably more studies have explored the relationship from earlier phonological awareness to later reading while the possibility of a bi-directional relationship has been considerably less well researched. It remains an unresolved issue if phonological awareness is a precursor skill for reading or if it develops as part of the process of learning to read (Bradley and Bryant, 1985; Castles and Coltheart, 2004; Hulme et al., 2005). Our study provided support for a bi-directional relationship between phonological awareness and reading but with a slightly stronger relationship between early phonological awareness to later reading. The finding is in line with past studies where phonological awareness has been found to be a robust predictor of reading (Lonigan et al., 2000; Ehri et al., 2001), and where reading difficulty (i.e., dyslexia) has been linked to deficits in phonological awareness (Lyon et al., 2003). Similarly, in a study by Hogan et al. (2005), there was a reciprocal relationship found between phonological awareness and word reading.

Overall, and in contrast to Chu et al. (2016), we observed a trend for domain-specific skills to be more strongly related to achievement at the end of kindergarten than at the beginning of kindergarten. While our analytical approach differs substantially from that in Chu et al. (2016), it is also probable that differences relate to the developmental stage that is evaluated. Children in our study were older and (consequently) had a wider range of mathematical abilities. The increasing differentiation of math and reading domains over development, as well as the fact that cross-domain associations seem more likely at earlier stages in development, is also consistent with findings from studies that have tracked the development of math and reading skills separately. For instance, Lee and Bull (2016) found that the role of prior mathematics achievement increased across grades from kindergarten to Grade 9.

### Limitations

Findings from this study should be considered in the context of several limitations. First, the use of the number sets task has its drawbacks, as fluency in identifying and processing quantities represented by numerals and object sets is not exactly a rudimentary skill compared to other early skills associated with mathematics, such as numeral recognition or numerical magnitude discrimination. Thus, there is a possibility that the use of measures of other types of basic skills involved in mathematics may have led to different results. A similar concern can be made regarding the reliance on phonological awareness as the measure of emerging literacy skill. Notably, phonological awareness is only one aspect of phonological process, which includes other components, such as phonological (verbal) working memory, that have also been reliably found to be a precursor for reading (Baddeley et al., 1998; see Baddeley, 2003 for a review). Additionally, other aspects of phonological processing, such as rapid automatized naming, which is pertinent in reading fluency, which have been found to be a longitudinal predictor of reading (Landerl et al., 2019) should also be considered. Future research on the predictors of reading and mathematical ability should seek to consider incorporating a broader variety of these skills. This would be essential for attaining a more holistic understanding of the unique influences that each of these skills may have on reading and mathematics ability and how such skills jointly interact over development.

Second, the relative contribution of domain-specific and cross-domain variables will probably depend on how the learning outcome has been operationalized. We used a measure of general math ability (TEMA) that does not allow us to tease apart specific skills. However, some studies show that the relative contribution varies for skills, such as geometry and measurement, compared to other skills, such as magnitude comparisons (LeFevre et al., 2010). In terms of word reading, while an established measure of reading ability (i.e., WRAT-4) was utilized to assess reading ability using individual word stimuli, it is debatable whether single word reading is representative of skill use in an everyday context. It has been noted that reading in real-life situations often involves several words strung into sentences, which involves the sequential and simultaneous processing of visual and semantic information; these processes are not examined during the reading of solitary words (Zoccolotti et al., 2020).

Third, while the present study has tried to account for other domain-general predictors, such as non-verbal reasoning, that may be involved in the cross-domain association between early reading and mathematics, it is not comprehensive. Future studies should consider the inclusion of other important predictors, such as working memory, where robust relationships have been found. In terms of math, decades of research have shown that working memory skills are closely related to math achievement and precursors of math, such as counting (Bull and Scerif, 2001; Bull et al., 2008; Monette et al., 2011; Van der Ven et al., 2012), as well as early numerical magnitude skills (Geary et al., 2009; Kolkman et al., 2013). Similarly, research indicates that both the phonological loop (verbal working memory) and the central executive are pertinent at different stages of reading. The phonological loop plays a crucial role in the early stages of reading where children start learning the concept of mapping of grapheme-phoneme and gain mastery of decoding, which facilitates both word and non-word reading (Baddeley, 2003). As children’s reading development progresses, the central executive is found to play a more important role in facilitating reading comprehension (Cain et al., 2004).

Finally, moderation effects may impact on the relative importance of within- *versus* cross-domain prediction. For instance, children in our sample who are higher achieving will be progressing to problems in the TEMA with increased task difficulty, e.g., word problem solving, which requires storing of verbal information without external support. These tasks may then require children to draw on their language and comprehension skills in solving these problems. This is consistent with previous studies (e.g., Purpura and Logan, 2015) that have found than domain-specific skills, such as processing of non-symbolic quantity, were more likely to predict performance of children at the lower end of the distribution who are completing only simple math questions, while performance of children at the upper end of the distribution (who are completing questions that required more translation between different representations) was more likely to be predicted by math language.

### Conclusion

This study is one of only a few to investigate the cross-domain associations between reading, mathematics, and their domain-specific predictors. Collecting data on all tasks across 3years have allowed us to examine the changing nature of the relationships within and across domains of learning. Path model analyses highlighted that the reciprocal relationship between reading and mathematics changes over time; specifically, that reading has a stronger influence on mathematics closer to the formal schooling years. The results indicated that math development is supported *via* two routes; firstly, a linguistic route that likely supports skills, such as mastery of numeral recognition and counting. Secondly, a quantitative route that supports children’s ability to accurately and fluently process and operate on quantity representations. These align with two of the three pathways to mathematics identified by LeFevre et al. (2010). Importantly, the findings revealed that phonological awareness and number sets fluency do not have a cross-domain effect on the later development of mathematics and reading, respectively. The findings have implications for the timing and nature of interventions focused on improving math and/or reading skills. Specifically, interventions for improving early reading and math abilities may consider tapping on the cross-domain association between these abilities if targeted at very young children. However, these interventions should generally seek to target domain-specific cognitive skill(s) with an established link to reading or math ability. Lastly, our findings also underscore the close link between reading and math during the first years and the progressive differentiation of each domain upon entry to formal school. This suggests that the pattern of reading and math disabilities may vary over development in the sense that the prevalence of comorbidity of reading and math disabilities would be higher in younger children—even if these children do not have a multifaceted deficit. In the same vein, our findings suggest examining how math and reading abilities jointly unfold to differentiate children with specific reading or math disabilities from those with more entrenched deficits.

## Data Availability Statement

The original contributions presented in the study are included in the article/Supplementary Material, and further inquiries can be directed to the corresponding author.

## Ethics Statement

The studies involving human participants were reviewed and approved by the National Institute of Education (NIE), Nanyang Technological University, Singapore. Written informed consent to participate in this study was provided by the participants’ legal guardian/next of kin.

## Author Contributions

FK: conceptualization, methodology, formal analysis, data curation, writing-original draft, writing-review and editing, visualization, and project administration. RB: conceptualization, formal analysis, writing-original draft, supervision, project administration, and funding acquisition. DM: conceptualization, methodology, formal analysis, writing-original draft, writing-review and editing, and visualization. All authors contributed to the article and approved the submitted version.

## Funding

This study was funded by the Singapore Ministry of Education (MOE) under the Education Research Funding Programme (OER 09/14 RB) and administered by the National Institute of Education (NIE), Nanyang Technological University, Singapore. Any opinions, findings, and conclusions or recommendations expressed in this material are those of the author(s) and do not necessarily reflect the views of the Singapore MOE and NIE.

## Conflict of Interest

The authors declare that the research was conducted in the absence of any commercial or financial relationships that could be construed as a potential conflict of interest.

## Publisher’s Note

All claims expressed in this article are solely those of the authors and do not necessarily represent those of their affiliated organizations, or those of the publisher, the editors and the reviewers. Any product that may be evaluated in this article, or claim that may be made by its manufacturer, is not guaranteed or endorsed by the publisher.

## Footnotes

^1^Average-measurement, consistency, and two-way mixed-effects model.
