# Supplementary material for: Cross- and Within-Domain Associations of Early Reading and Mathematical Skills: Changes Across the Preschool Years
Source: Front Psychol. 2021 Oct 12;12:710470. doi: 10.3389/fpsyg.2021.710470 (PMC8546213; doi:10.3389/fpsyg.2021.710470)
Supplement: Supplementary file 1 [file Table_1.DOCX]

Table S1: Standardized parameter estimates (and SE) of the associations between domain-general aspects and reading/math aspects over the kindergarten years.

|  | **SES** | **NvIn** | **Age entry to K1** |
| --- | --- | --- | --- |
| Math1 | 0.249*** (0.042) | 0.356*** (0.04) | 0.191*** (0.038) |
| Math2 | 0.117*** (0.03) | 0.046 (0.031) | - |
| Math3 | 0.038 (0.031) | 0.005 (0.029) | - |
| Nset1 | 0.087* (0.045) | 0.303*** (0.041) | 0.191*** (0.041) |
| Nset2 | 0.116** (0.035) | 0.131*** (0.035) | - |
| Nset3 | -0.018 (0.037) | 0.089* (0.037) | - |
| Read1 | 0.251*** (0.044) | 0.24*** (0.041) | 0.106** (0.038) |
| Read2 | 0.108** (0.032) | -0.022 (0.033) | - |
| Read3 | -0.026 (0.03) | 0.013 (0.031) | - |
| Phaw1 | 0.298*** (0.042) | 0.245*** (0.044) | 0.181*** (0.041) |
| Phaw2 | 0.172*** (0.034) | 0.012 (0.036) | - |
| Phaw3 | 0.037 (0.039) | 0.026 (0.038) | - |
